# Public Confidence in Non-COVID Vaccines: Influenza, Human Papillomavirus (HPV), and Childhood Immunizations in the Western Region of Saudi Arabia

**DOI:** 10.7759/cureus.83740

**Published:** 2025-05-08

**Authors:** Doaa S Baashar, Jihad A Malibary, Sultan S Alam, Abdulaziz A Suwaidi, Almuhanad A Alyami, Amjad A Marouf, Mokhtar Shatla

**Affiliations:** 1 Medical School, Umm Al-Qura University, Makkah, SAU; 2 Family Medicine, Ministry of Health Holdings, Jeddah, SAU; 3 Family Medicine, Al-Thagher Hospital, Ministry of Health, Jeddah, SAU; 4 General Practice, Al-Thagher Hospital, Ministry of Health, Jeddah, SAU; 5 Family Medicine, College of Medicine, Umm Al-Qura University, Makkah, SAU

**Keywords:** covid-19, immunization, saudi arabia, vaccine confidence, vaccine trust, western region

## Abstract

Background

The COVID-19 pandemic has significantly affected global attitudes toward vaccines. Although COVID-19 vaccines have proven to be both effective and safe, they continue to face hesitancy and misinformation. The current study assessed the impact of the pandemic on trust in routine vaccines, offering insights to improve vaccine acceptance and coverage. Moreover, the study identified the impact of demographic and socio-economic factors on vaccine confidence in Western Saudi Arabia.

Methods

This observational cross-sectional online survey study was carried out between January and February 2025, focusing on adult individuals in Western Saudi Arabia. Data collection involved a revised questionnaire, which was adapted from a previously validated version through Google Forms (Google, Mountain View, CA) online and included responses from participants' demographic information, the Vaccine Trust Gauge, and questions about the COVID-19 vaccine. Statistical analyses were conducted using IBM SPSS Statistics, Version 26 (IBM Corp., Armonk, NY). A descriptive analysis was utilized to summarize categorical data through the use of counts and percentages. Fisher's Exact test and multinomial logistic regression assessed the relationship between demographic characteristics and COVID-19 vaccine perspectives and vaccine trust levels. A significance level of 0.05 was established as the threshold for statistical significance.

Results

Out of 373, 195 (52.3%) of the participants were female individuals. The age distribution revealed that 134 (35.9%) were between 21 and 30, while 124 (33.2%) fell within the 31-40 age range. The majority of participants were Saudi nationals, 351 (94.1%), and not healthcare workers, 296 (79.4%). Additionally, 207 (55.5%) participants held bachelor’s degrees. A significant portion of the participants demonstrated a high level of vaccine trust, with 296 (79.4%) expressing confidence in the vaccine, such as influenza, human papillomavirus (HPV), and childhood immunizations. Most participants were aware of the COVID-19 vaccine (97.6%). The study identified significant impacts of demographic characteristics on vaccine trust levels by using Fisher's Exact test. Age (p=0.031), gender (p=0.046), and nationality (p=0.006) all showed notable differences. Furthermore, perspectives on the COVID-19 vaccine significantly influenced vaccine trust levels (p<0.001), indicating strong statistical significance.

Conclusion

Our study found high vaccine trust in Western Saudi Arabia, with younger participants, Saudi nationals, and female individuals showing the most trust. The perception of COVID-19 vaccines significantly influenced overall vaccine trust, highlighting the need for effective communication about vaccine safety. These findings suggest that addressing demographic differences and specific concerns can enhance vaccine trust and uptake.

## Introduction

Vaccination has significantly influenced global health; its effects are still noteworthy today, from the successful elimination of smallpox in 1979 to the efforts aimed at minimizing the COVID-19 risk in 2020 [1,2]. To safeguard against avoidable infectious diseases, the Centers for Disease Control and Prevention (CDC) advises that all adults obtain specific vaccinations tailored to their age and health status. This includes the annual influenza vaccine for Individuals older than six months and pneumococcal disease, particularly adults who have certain risk factors, such as smokers or those aged 65 and older [3]. The Expanded Program of Immunization by the World Health Organization (WHO), along with the Global Alliance for Vaccines and Immunization (GAVI), has played a crucial role in enhancing vaccination rates worldwide against various infectious diseases [4,5]. GAVI acknowledges the proven efficacy of the human papillomavirus (HPV) vaccine in reducing the rates of cervical cancer. Consequently, it is advised that routine HPV vaccinations be administered to children between the ages of 11 and 12, although it can begin as early as nine and continue up to the age of 26 [3].

Confidence in the safety and effectiveness of vaccines is crucial for the success of global immunization initiatives [6]. Trust is fundamental to vaccine acceptance, influencing individual health outcomes and the overall public health environment. Vaccine confidence encompasses more than just personal assurance regarding a vaccine's safety and effectiveness; it also involves faith in the organizations responsible for its regulation, development, and distribution. The connection between how vaccine quality is perceived and its associated safety and the trustworthiness of the institutions supporting the vaccine greatly affects an individual's decision to get vaccinated [7].

Although vaccines have significantly reduced the incidence of fatal diseases and Saudi Arabia’s obligatory vaccination program ensures high immunization coverage, continued efforts are essential to enhance public awareness, understanding, and voluntary acceptance of vaccination initiatives [8]. A 2021 study on Saudi public perceptions of seasonal influenza vaccination surveyed 790 individuals aged 15 and older [9]. Only 12.65% reported regular vaccination, with the highest rate (57%) among those under 24. Over 90% of participants with chronic conditions had inconsistent vaccination histories for influenza. Those who perceived the vaccine as safe and necessary were more likely to get vaccinated, highlighting a low vaccination rate in the population [9]. A study in 2022 involving 13,280 Saudi adults assessed their knowledge, attitudes, and practices (KAP) regarding the pneumococcal vaccine [10]. The results showed that 65.7% had poor knowledge, over half had a positive attitude, and 43.7% reported incorrect practices. Higher KAP levels were linked to adult males with higher education and those working in healthcare, indicating limited knowledge but a generally positive attitude toward the vaccine [10]. Moreover, research in the Gulf Cooperation Council countries shows that despite high overall vaccine confidence, concerns about safety and side effects impact vaccine acceptance, including non-COVID vaccines [11]. It highlights that distrust in vaccine safety plays a significant role in hesitancy [11]. This emphasizes the need for effective public health messaging and cultural and religious endorsements to build trust in vaccines [11]. 

Recent estimates from the World Health Organization (WHO) and UNICEF indicate that routine immunization coverage in Saudi Arabia remains consistently high [12-13]. For instance, key vaccines included in the national immunization schedule-such as the diphtheria-tetanus-pertussis (DTP3) and measles-containing vaccines-achieve coverage rates that typically exceed 90% and often approach or surpass 95% among the targeted pediatric populations. This impressive coverage can be attributed to the Kingdom’s well-established mandatory vaccination program and its robust public health infrastructure, both of which ensure effective vaccine delivery and adherence to immunization schedules. These statistics highlight the effectiveness of Saudi Arabia’s immunization policies, while also emphasizing the need for ongoing vigilance through continuous monitoring and targeted public education to address any emerging concerns or gaps in awareness [12-13].

Research has indicated that individuals who perceive a greater risk of contracting COVID-19, recognize the seriousness of its complications, and believe in the benefits of vaccination are more inclined to get vaccinated. On the other hand, those who express doubts regarding the effectiveness and potential side effects of COVID-19 vaccines tend to have lower vaccination intentions [14]. As the world continues to face the challenges brought on by COVID-19, it is crucial to explore the factors influencing vaccine confidence, as this understanding not only pertains to COVID-19 but can also affect public attitudes toward other respiratory vaccines like the flu shot. A recent study has found that the pandemic has heightened the willingness to receive flu vaccinations [15]. Conversely, there are also indications that healthcare workers have shown a decline in flu vaccination rates during the COVID-19 pandemic, which may be attributed to the focus on COVID-19 vaccine campaigns overshadowing the importance of flu vaccination or as a result of vaccine fatigue [16].

The COVID-19 pandemic has notably changed public perceptions and behaviors regarding routine vaccinations beyond just COVID-19 vaccines. This presents an opportunity to reassess how these perceptions have evolved and to identify factors influencing vaccine confidence. Understanding public trust in non-COVID-19 vaccines is essential for maintaining high vaccination rates and public health. This study aimed to examine the pandemic's impact on trust in routine vaccines in Makkah, offering insights to improve vaccine acceptance and coverage.

## Materials and methods

Setting and design

This study was a cross-sectional online survey utilizing a self-administered questionnaire conducted among adults in Western Saudi Arabia.

Study subjects

Our study population included adults from the Western region of Saudi Arabia. The criteria for inclusion required participants to be at least 15 years old, regardless of gender. Individuals younger than 15 years were excluded from the study, as were those who refused to participate.

Data collection

Data collection for the research was carried out from January to February 2025. The method employed was a questionnaire through an online Google Forms (Google, Mountain View, CA).

Data collection tool

The online survey for this study was created using a questionnaire that had been validated in prior research [17] in similar settings culture and was divided into three sections: (1) demographic information, (2) the Vaccine Trust Gauge, and (3) questions specifically about the COVID-19 vaccine. The demographic section gathers age, gender, educational background, and average annual income. The Vaccine Trust Gauge consists of questions designed to evaluate overall vaccine trust, demonstrating high internal consistency. Additionally, the survey includes questions regarding attitudes toward COVID-19 vaccines, formulated following suggestions from experts in public health and infectious diseases. This comprehensive approach to instrument validation, encompassing psychometric properties and context-specific content, ensures that the tool is both reliable and valid for assessing vaccine trust in the Saudi Arabian setting.

Sample size calculation

The sample size determination was carried out utilizing the Raosoft (Raosoft Inc., Seattle, WA) online calculator. A margin of error of 5% was established, with a 90% confidence level, along with the maximum uncertainty. Additionally, the study must encompass at least 267 adult individuals from Saudi Arabia.

Scoring of the questionnaire

The Vaccine Trust Gauge scores were compiled and transformed to a scale ranging from 0 to 1, where 0 indicates a total lack of trust and 1 signifies full trust. These scores were then classified into three levels of trust (high, medium, and low) using intervals of 0.33. Opinions on the COVID-19 vaccine were categorized into three groups: agree (including both strongly agree and somewhat agree), neutral, and disagree (which includes strongly disagree and somewhat disagree).

Statistical analysis

The information was logged into a Microsoft Excel 16 (Microsoft Corp., Redmond, WA)) spreadsheet for evaluation and subsequently imported into IBM SPSS Statistics, Version 26 (IBM Corp., Armonk, NY) for further analysis. A descriptive analysis was conducted to summarize categorical data by utilizing numerical values and percentages. Fisher's Exact test and multinomial logistic regression assessed the relationship between demographic characteristics and COVID-19 vaccine perspectives and vaccine trust levels. A significance level of 0.05 was used as the threshold for statistical significance. The percentage calculated accounted for any missing data points.

Ethical considerations

Approval for the study was secured from the Institutional Review Board (IRB) at Umm Al-Qura University (approval no. HAPO-02-K-012-2025-03-2593) prior to commencing the research. An introductory statement was included in the questionnaire to explain that the study was being conducted for research purposes, detailing its objectives and the intended participants. This statement emphasized that participation was entirely voluntary, with data being gathered without revealing identities. Every detail shared by the participants was treated as confidential and remained within the group conducting the study. No identifying details or names were recorded.

## Results

A total of 373 individuals took part in our research, most within the age range of 21-30 years (134, 35.9%) and 31-40 years (124, 33.2%). Most were Saudi nationals (351, 94.1%). About half were male individuals (195, 52.3%), married (182, 48.8%), and had Bachelor's degrees (207, 55.5%). About two-thirds were employed (244, 65.4%), and most were not healthcare workers (296, 79.4%). Regarding income, 165 (44.2%) of the earnings are between 10,000 and 20,000 SAR (Saudi Arabian Riyals) per month. Geographically, participants were primarily from Makkah (116, 31.1%) and Jeddah (105, 28.2%), with other regions accounting for 127 (34.0%), as shown in Table 1.

**Table 1:** Demographics among adults in Saudi Arabia (n=373) Values are presented as N (%) SAR: Saudi Arabian Riyals

Factors	Category	Number	Percentage
Age	15-20	15	4.0
	21-30	134	35.9
	31-40	124	33.2
	41-50	55	14.7
	51-60	34	9.1
	>60	11	2.9
Gender	Female	178	47.7
	Male	195	52.3
Nationality	Saudi	351	94.1
	Non-Saudi	22	5.9
Education level	Illiterate	17	4.6
	Primary school	9	2.4
	High school	113	30.3
	Bachelor's degree	207	55.5
	Postgraduate degree (PhD or Master's)	27	7.2
Employment	Employed	244	65.4
	Not employed	129	34.6
Healthcare worker	Yes	77	20.6
	No	296	79.4
Marital status	Single	150	40.2
	Married	182	48.8
	Widowed	11	2.9
	Divorced	30	8.0
Monthly income (SAR)	Less than 10,000	126	33.8
	10,000-20,000	165	44.2
	More than 20,000	82	22.0
Resident region	Makkah	116	31.1
	Jeddah	105	28.2
	Taif	25	6.7
	Other	127	34.0

Table 2 presents the Vaccine Trust Gauge among participants in Saudi Arabia. Over half of the participants (214, 57.4%) express a high level of trust in the safety of vaccines, while 148 (39.7%) believe that vaccines effectively protect them from serious illness. Furthermore, 146 (39.1%) strongly trust that vaccines pose a lower risk to their health, and 134 (35.9%) agree that the benefits outweigh the known or potential risks. Additionally, 154 (41.3%) of participants are confident that vaccines, including influenza, HPV, and childhood immunizations, are effective.

**Table 2:** Vaccine Trust Gauge among participants in Saudi Arabia (n=373) Values are presented as N (%) HPV: human papillomavirus

Thinking about different vaccines recommended to you as an adult, how much trust do you have in the following five statements?		A great deal of trust	A lot of trust	A little trust	No trust at all
1.	Vaccines are safe, such as influenza, HPV, and childhood immunizations	214 (57.4)	56 (15.0)	81 (21.7)	22 (5.9)
2.	Vaccines protect you from serious illness	148 (39.7)	154 (41.3)	60 (16.1)	11 (2.9)
3.	Vaccines are a lower risk to your health (in terms of a possible bad outcome) than the diseases they prevent	146 (39.1)	116 (31.1)	91 (24.4)	20 (5.4)
4.	The benefits of vaccines are greater than the known or potential risks of vaccines.	134 (35.9)	115 (30.8)	81 (21.7)	43 (11.5)
5.	Vaccines are effective, such as influenza, HPV, and childhood immunizations	154 (41.3)	97 (26.0)	85 (22.8)	37 (9.9)
Thinking about vaccines in general		Strongly agree	Somewhat agree	Somewhat disagree	Strongly disagree
6.	Vaccines are important for children to have	291 (78.0)	56 (15.0)	20 (5.4)	6 (1.6)
7.	Vaccines are important for adults to have	190 (50.9)	103 (27.6)	54 (14.5)	26 (7.0)
Different people and/or organizations make vaccine recommendations for adults. Kindly indicate how much confidence you have in their suggestions regarding vaccines:		A great deal of trust	A lot of trust	A little trust	No trust at all
8.	Recommendations by a National Public Health Agency	207 (55.5)	85 (22.8)	55 (14.7)	26 (7.0)
9.	Recommendations from my healthcare provider (e.g., doctor or pharmacist)	151 (40.5)	141 (37.8)	60 (16.1)	21 (5.6)
10.	Recommendations from my state or local health department	193 (51.7)	89 (23.9)	73 (19.6)	18 (4.8)
11.	Recommendation from my employer	51 (13.7)	71 (19.0)	123 (33.0)	128 (34.4)
12.	Recommendations from my close friends and family	79 (21.1)	65 (17.4)	112 (30.0)	117 (31.4)

A significant majority of people recognize the importance of vaccines, with 291 (78.0%) believing they are crucial for children and 190 (50.9%) for adults. Trust in vaccine recommendations varies, with 207 (55.5%) trusting national public health agencies and 151 (40.5%) trusting healthcare providers. Additionally, 193 (51.7%) have a great deal of trust in their state or local health departments. In contrast, only 51 (13.7%) trust recommendations from their employers, and 79 (21.1%) trust advice from friends and family. A majority of participants in Saudi Arabia have a high level of vaccine trust (296, 79.4%).

Table 3 provides insights into the perceptions of the COVID-19 vaccine held by individuals in Saudi Arabia. Nearly half (183, 49.1%) of participants express high concern about illness caused by COVID-19. Additionally, 96 (25.7%) strongly agree on the effectiveness of COVID-19 vaccines, while 69 (18.5%) believe they are safe. Regarding trust in the science behind the vaccines, 70 (18.8%) of participants express strong trust. Regarding booster shots, 63 (16.9%) are committed to continuing them if recommended, and 75 (20.1%) emphasize the importance of matching boosters to current variants. Finally, 81 (21.7%) of participants strongly prefer traditional vaccines over mRNA vaccines. Most participants in Saudi Arabia recognized the COVID-19 vaccine, with awareness reported at 364 (97.6%).

**Table 3:** Perceptions of the COVID-19 vaccine among individuals in Saudi Arabia Values are presented as N (%)

COVID-19 vaccine perceptions		Strongly agree	Somewhat agree	Neutral/no opinion	Somewhat disagree	Strongly disagree
1.	Concerned about COVID-19 illness	183 (49.1)	70 (18.8)	67 (18.0)	38 (10.2)	15 (4.0)
2.	Vaccines for COVID-19 demonstrate significant efficacy	96 (25.7)	134 (35.9)	79 (21.2)	41 (11.0)	23 (6.2)
3.	The vaccines for COVID-19 are considered to be safe	69 (18.5)	78 (20.9)	134 (35.9)	57 (15.3)	35 (9.4)
4.	Have faith in the research supporting the efficacy of COVID-19 vaccines	70 (18.8)	63 (16.9)	94 (25.2)	103 (27.6)	43 (11.5)
5.	Continue to obtain the vaccine for COVID-19 boosters if recommended	63 (16.9)	42 (11.3)	104 (27.9)	75 (20.1)	89 (23.9)
6.	It’s essential for the booster vaccine I get to align with the variants that are currently spreading	75 (20.1)	61 (16.4)	108 (29.0)	79 (21.2)	50 (13.4)
7.	Trust traditional vaccines compared to mRNA vaccines	81 (21.7)	55 (14.7)	144 (38.6)	58 (15.5)	35 (9.4)

Table 4 highlights the significant impact of demographic characteristics on vaccine trust levels, with age (p= 0.031), gender (p= 0.046), and nationality (p= 0.006) all showing significant differences. Participants aged 15-30 exhibited a higher vaccine trust level (128, 85.9%) than those older than 30 (168, 75%). Saudi nationals had a higher trust level (280, 79.8%) compared to non-Saudis (16, 72.7%). Additionally, female individuals (141, 79.5%) demonstrated more trust in vaccines than male individuals (155, 79.2%). After applying multinomial logistic regression, age (p=0.002), (p=0.004), and income (p=0.019) were the significant factors.

**Table 4:** Impact of demographic characteristics on vaccine trust level Values are presented as N (%), significant p-values are marked with an *, p-value considered significant at p<0.05 SAR: Saudi Arabian Riyals

Factors	Category	Vaccine trust level number (percentage)			p-value
		High	Medium	Low	
Age	15-30	128 (85.9)	20 (13.4)	1 (0.7)	0.031*
	>30	168 (75.0)	51 (22.8)	5 (2.2)	
Gender	Female	141 (79.2)	37 (20.8)	0 (0)	0.046*
	Male	155 (79.5)	34 (17.4)	6 (3.1)	
Nationality	Saudi	280 (79.8)	68 (19.4)	3 (0.9)	0.006*
	Non-Saudi	16 (72.7)	3 (13.6)	3 (13.6)	
Education level	High school or less	112 (80.6)	24 (17.3)	3 (2.2)	0.679
	Bachelor's degree or above	184 (78.6)	47 (20.1)	3 (1.3)	
Employment	Employed	200 (82.0)	42 (17.2)	2 (0.8)	1
	Not employed	96 (74.4)	29 (22.5)	4 (3.1)	
Healthcare worker	Yes	64 (83.1)	13 (16.9)	0 (0)	0.5
	No	232 (78.4)	58 (19.6)	6 (2.0)	
Marital status	Married	142 (78.0)	38 (20.9)	(1.1)	0.516
	Unmarried	154 (80.6)	33 (17.3)	4 (2.1)	
Monthly income (SAR)	Less than 10,000	93 (73.8)	29 (23.0)	4 (3.2)	0.113
	10,000-20,000	131 (79.4)	32 (19.4)	2 (1.2)	
	More than 20,000	72 (87.8)	10 (12.2)	0 (0)	
Resident region	Makkah	86 (74.1)	27 (23.3)	3 (2.6)	0.367
	Jeddah	87 (82.9)	16 (15.2)	2 (1.9)	
	Other	123 (80.9)	28 (18.4)	1 (0.7)	

Table 5 illustrates the COVID-19 vaccine impact perspectives on vaccine trust level, with significant differences observed (p< 0.001). Individuals who believe that COVID-19 vaccines are effective show a high trust level of 211 (91.7%), while those who are neutral or disagree have significantly lower high trust levels at 58 (73.4%) and 27 (42.2%), respectively. Similarly, 142 (96.6%) of those who approve that COVID-19 vaccines are safe have high trust, compared to 103 (76.9%) of neutral and 51 (55.4%) of those who disagree.

**Table 5:** Impact of COVID-19 vaccine perspectives on vaccine trust level Values are presented as N (%), significant p-values are marked with an *, p-value considered significant at p<0.05

Factors	Category	Vaccine trust level number (percentage)			p-value
		High	Medium	Low	
Awareness of COVID-19 vaccine	Yes	290 (79.7)	68 (18.7)	6 (1.6)	0.414
	No	6 (71.4)	2 (28.6)	0 (0)	
	Unsure	1 (50.0)	1 (50.0)	0 (0)	
Concerned about COVID-19 illness	Agree	209 (82.6)	41 (16.2)	3 (1.2)	0.112
	Neutral	50 (74.6)	16 (23.9)	1 (1.5)	
	Disagree	37 (69.8)	14 (26.4)	2 (3.8)	
Vaccines for COVID-19 demonstrate significant efficacy	Agree	211 (91.7)	19 (8.3)	0 (0)	<0.001*
	Neutral	58 (73.4)	21 (26.6)	0 (0)	
	Disagree	27 (42.2)	31 (48.4)	6 (9.4)	
The vaccines for COVID-19 are considered to be safe	Agree	142 (96.6)	5 (3.4)	0 (0)	<0.001*
	Neutral	103 (76.9)	31 (23.1)	0 (0)	
	Disagree	51 (55.4)	35 (38.0)	6 (6.5)	
Have faith in the research supporting the efficacy of COVID-19 vaccines	Agree	126 (94.7)	7 (5.3)	0 (0)	<0.001*
	Neutral	72 (76.6)	22 (23.4)	0 (0)	
	Disagree	98 (67.1)	42 (28.8)	6 (4.1)	
Continue to obtain the vaccine for COVID-19 boosters if recommended	Agree	101 (96.2)	4 (3.8)	0 (0)	<0.001*
	Neutral	84 (80.8)	20 (19.2)	0 (0)	
	Disagree	111 (67.7)	47 (28.7)	6 (3.7)	
It’s essential for the booster vaccine I get to align with the variants that are currently spreading	Agree	128 (94.1)	8 (5.9)	0 (0)	<0.001*
	Neutral	81 (75.0)	27 (25.0)	0 (0)	
	Disagree	87 (67.4)	36 (27.9)	6 (4.7)	
Trust traditional vaccines compared to mRNA vaccines	Agree	11 (8.1)	125 (91.9)	0 (0)	<0.001*
	Neutral	33 (22.9)	110 (76.4)	1 (0.7)	
	Disagree	27 (29.0)	61 (65.6)	(5.4)	

Having confidence in the scientific research supporting COVID-19 vaccinations also affects trust levels: 126 (94.7%) of those who agree show high trust, whereas only 72 (76.6%) of neutral and 98 (67.1%) of those who disagree exhibit high trust. The willingness to continue getting boosted if recommended is associated with a high trust level of 101 (96.2%) among those who agree, compared to 84 (80.8%) of neutral and 111 (67.7%) of those who disagree. Furthermore, 128 (94.1%) of participants who agree that boosters should match current variants show high trust, while only 81 (75.0%) of neutral and 87 (67.4%) of those who disagree do. On the other hand, participants who trust traditional vaccines more than mRNA vaccines have a lower high trust level (11, 8.1%) compared to neutral (33, 22.9%) and disagree (27, 29.0%). After applying multinomial logistic regression, concern about illness caused by COVID-19 was the only significant item (p<0.001).

## Discussion

Our study found a generally high level of trust in vaccines among participants in Western Saudi Arabia, with significant demographic factors impacting these trust levels. Younger participants (aged 15-30 years), Saudi nationals, and female individuals showed higher levels of trust in vaccines compared to older individuals, non-Saudis, and male individuals. People were not sufficiently informed about infectious diseases and the critical role of vaccinations in Saudi Arabia. A study in Al Ahsa revealed significant gaps in vaccine-related knowledge: 31% believed vaccinations should be avoided during pregnancy, 40.2% did not see the flu vaccine as essential for pregnant women, and 40.4% were unaware of its importance [18]. Moreover, 61.7% had not participated in any conversations regarding vaccination while pregnant. Although 59% expressed willingness to participate in educational activities [18]. Furthermore, another study in Saudi Arabia revealed that only 34.5% recognized HPV infections, and awareness of its link to cervical cancer stood at 27.4% [19]. Awareness of the HPV vaccine was low at 32.3%, with higher levels noted among non-Saudis and guardians compared to participants. A total of 64.3% of participants in the research expressed their willingness to get vaccinated [19].

Our results align with earlier studies conducted in Makkah, Saudi Arabia, which identified that higher educational attainment, female gender, and prior exposure to COVID-19 serve as predictors of willingness to receive vaccinations and confidence in their efficacy [20]. Additionally, another study highlighted high levels of vaccine trust in various countries, with Brazil at 78.8%, India at 66.7%, Nigeria at 61.8%, and the UK at 60.8% [17]. The study also indicated that higher trust levels are typically found among younger participants, those with higher education, and individuals with above-average incomes [17].

Moreover, our study showed high awareness regarding the COVID-19 vaccine in Western Saudi Arabia. Similarly, a study showed a good understanding of COVID-19 immunization in Makkah [20]. Various studies have examined the level of public awareness regarding COVID-19 vaccinations in Egypt [21], Vietnam [22], Libya [23], and Ethiopia [24]. In contrast, the overall level of knowledge in India was found to be poor, with nearly half of the participants reporting incorrect answers or expressing uncertainty [25].

Individual perceptions of the COVID-19 vaccine significantly impact the shaping of overall vaccine trust. Participants who believed in the effectiveness and safety of COVID-19 vaccines exhibited significantly higher trust levels in vaccines overall. This is consistent with a study conducted at King Abdulaziz University, Jeddah, which emphasized that individuals' perceptions of health and their confidence in the sources of health information played a crucial role in their openness to getting vaccinated against COVID-19 [26]. Moreover, another study showed that participants who had a strong trust in vaccines tended to express greater confidence in the COVID-19 vaccine, regardless of their country of origin [17]. Significant associations (p<0.001) were found between vaccine trust levels and concerns about illness caused by COVID-19, belief in the effectiveness of the COVID-19 vaccine, agreement on the safety of COVID-19 vaccines, trust in the science behind COVID-19 vaccines, and preference for traditional vaccines over mRNA vaccines [17]. Our findings also revealed that some participants strongly prefer traditional vaccines over mRNA vaccines.

Additionally, our study identified the need for increased trust in recommendations from healthcare providers and public health agencies. While most participants trusted national public health agencies, a notable portion still relied on other sources of information. This finding is consistent with research that highlighted the importance of institutional trust in vaccine acceptance [27]. Therefore, targeted communication strategies, educational initiatives, and strong advocacy by healthcare professionals and public health authorities are essential in building and maintaining high levels of vaccine trust in the population. Future research should continue to explore the factors influencing vaccine trust and develop tailored interventions to address specific concerns and trust gaps among different demographic groups.

Limitations

The research had limitations due to its cross-sectional approach, which heightened the likelihood of selection bias, as participants were frequently selected according to their readiness or eagerness to participate. The self-administered questionnaire could have led to response bias as participants may have provided inaccurate answers due to social pressure, potentially skewing their perceptions. Additionally, confounding bias may have occurred due to the lack of accounting for all factors affecting vaccine trust levels.

## Conclusions

Our study revealed a generally high level of vaccine trust among participants in Western Saudi Arabia, with significant differences based on demographic characteristics. Younger participants, Saudi nationals, and females exhibited higher trust levels. The perception of COVID-19 vaccines also greatly influenced overall vaccine trust, highlighting the importance of effective communication and education about vaccine safety and efficacy. These findings emphasize the need to address specific concerns and demographic differences to improve vaccine trust and uptake.
